# Poliovirus excretion following vaccination with live poliovirus vaccine in patients with primary immunodeficiency disorders: clinicians’ perspectives in the endgame plan for polio eradication

**DOI:** 10.1186/s13104-018-3822-7

**Published:** 2018-10-11

**Authors:** Nermeen M. Galal, Safaa Meshaal, Rabab ElHawary, Eman Nasr, Laila Bassiouni, Humayun Ashghar, Noha H. Farag, Ondrej Mach, Cara Burns, Jane Iber, Qi Chen, Aisha ElMarsafy

**Affiliations:** 1 Department of Pediatrics, Cairo University, Cairo University Specialized Pediatric Hospital, 1 Ali Ibrahim Street, Mounira, Cairo, Egypt; 2 Department of Clinical and Chemical Pathology, Cairo University, 2 Ali Ibrahim Street, Kasr Alainy, Cairo, 11956 Egypt; 3 Holding Company for Biological Products and Vaccines, VACSERA, Regional Reference Polio Laboratory, 51 Wezaret ElZeraa, Al Agouzah, Dokki, Giza, 22311 Egypt; 4 World Health Organization, EMRO and HQ, Avenue Appia 20, 1202 Geneva, Switzerland; 5 Centers for Disease Control and Prevention, 1600 Clifton Road, Atlanta, GA 30333 USA

**Keywords:** Polio eradication, Immunodeficiency, Virus excretion

## Abstract

**Objective:**

Primary immunodeficiency (PID) patients are prone to developing viral infections and should not be vaccinated with live vaccines. In such patients, prolonged excretion and viral divergence may occur and they may subsequently act as reservoirs in the community introducing mutated virus and jeopardizing polio eradication. One hundred and thirty PID cases were included for poliovirus detection in stool with assessment of divergence of detected polioviruses from oral polio vaccine (OPV) virus. Clinical presentations of PID patients with detectable poliovirus in stool specimens are described.

**Results:**

Six PID patients (4.5%) had detectable vaccine-derived poliovirus (VDPV) excretion in stool specimens; of these, five patients had severe combined immunodeficiency (two with acute flaccid paralysis, one with meningoencephalitis and two without neurological manifestations), and one patient had X-linked agammaglobulinemia (paralysis developed shortly after diagnosis of immunodeficiency). All six case-patients received trivalent OPV. Five case-patients had type 2 immunodeficiency-related vaccine-derived polioviruses (iVDPV2) excretion; one had concomitant excretion of Sabin like type 3 virus and one was identified as iVDPV1 excretor. Surveillance for poliovirus excretion among PID patients is critical as these patients represent a potential source to reseed polioviruses into populations.

## Introduction

Primary immunodeficiency disorders (PIDs) are a group of diseases that mainly present with recurrent, severe or unusual infections. PIDs are underreported in developing countries because of lack of awareness. Given high rates of consanguinity in some countries, autosomal recessive disorders form the bulk of PIDs compared to Western populations. In Egypt, studies found that combined T cell and B-cell immunodeficiency disorders were more common than B-cell deficiency disorders alone [1, 2].

A study of poliovirus excretion among patients with PIDs from 13 OPV-using countries reported that patients with combined immunodeficiency (CID) disorders had an increased risk of delayed poliovirus clearance compared to patients with humoral deficiencies [3]. In Egypt, due to the absence of neonatal screening programs for PIDs, such patients often present to medical care after having received repeated OPV doses. Prolonged intestinal replication of OPV virus in PID patients can lead to development of vaccine-derived polioviruses (VDPV), which are referred to as immunodeficiency-related VDPV (iVDPV) [4]. VDPV excretors pose a threat to polio eradication efforts because they may continue to excrete and act as a reservoir in the community. They may develop acute flaccid paralysis (AFP) in various forms [5].

## Main text

### Methodology

Children seen at Cairo University with suspected PID diagnosed per the IUIS classification [6] were investigated after informed consent was obtained from their guardians. In addition, VDPV-excreting AFP case-patients, detected through the AFP surveillance system, were assessed using flow cytometric immunophenotyping of peripheral lymphocytes and immunoglobulin quantitation by nephelometry. Normal lymphocyte subsets were assessed according to age [7].

Two stool specimens, 24–48 h apart, were collected. The laboratory at VACSERA conducted poliovirus isolation, as well as intratypic differentiation (ITD) real-time PCR assays as per Global Polio Laboratory Network guidelines [8].

All detected polioviruses were genetically sequenced by the Polio and Picornavirus Laboratory, CDC. A VDPV was defined as a poliovirus isolate with at least ≥ 10 nucleotide differences in the VP1 region from Sabin strain for serotypes 1 and 3, and ≥ 6 differences for serotype 2 [9]. A Sabin-like (SL) virus was defined as virus related to Sabin vaccine virus with no mutations or lower number of nucleotide differences from Sabin than required for VDPV classification (< 10 nucleotide differences for poliovirus types 1 and 3 and < 6 for poliovirus type 2). If a VDPV or SL virus was detected, stool sampling was repeated monthly until either the sample was no longer positive on three occasions or the patient was lost to follow-up.

### Results

**Fig. 1:**
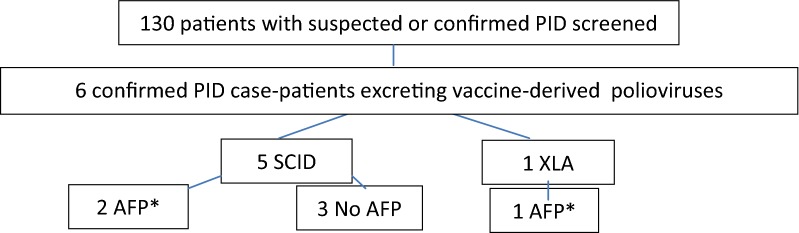
Detection of vaccine-derived poliovirus among patients with suspected or confirmed primary immunodeficiency (PID) disorders diagnosed at Cairo University Immunology Center, Egypt, 2011–2017. *PID* primary immune deficiency, *SCID* severe combined immunodeficiency, *XLA* X-linked agammaglobulinemia, *AFP* acute flaccid paralysis. *Patients presented with acute flaccid paralysis (AFP), were screened for poliovirus excretion and referred to Cairo University Immunology Center for PID screening. Seventh patient excreted SL3, no VDPV was isolated

One hundred and thirty patients with suspected or confirmed PID disorders were screened; six (4.6%) patients were excreting VDPV (Fig. 1).

- Three (2.3%) cases developed AFP following vaccination.
- Five cases of severe combined immunodeficiency disorder (SCID):
  - Two presented with AFP.
  - Two without neurological manifestations.
  - One presented with fever, seizures and altered consciousness (meningoencephalitis).
- One case of XLA (X-linked agammaglobulinemia) developed AFP.

All AFP case-patients received OPV vaccine. Five AFP case-patients were identified as iVDPV2 excretors (one patient had concomitant excretion of SL virus type 3) and one was identified as an iVDPV1 excretor.

A seventh MHC Class II deficiency patient developed AFP following OPV. She excreted SL3 virus and died a few weeks shortly after. No iVDPV excretion was detected in this patient until death.

### Patient 1

A 7-month-old female presented with acute pneumonia, diarrhea, failure to thrive and candidiasis. The patient had no evidence of neurological disease. Immunologic investigation identified absolute lymphopenia with low CDs and Immunoglobulins. A diagnosis of T-B− SCID due to RAG2 homozygous mutation [c.283G>A] was confirmed. An iVDPV2 was detected and viral shedding persisted for 5 months until death from pneumonia.

#### Patient 2

A 6-month-old female infant presented with recurrent oral thrush and unresolving pneumonia. Her family history was positive. Screening identified lymphopenia with low CDs and Immunoglobulins. A diagnosis of T-B− SCID was made, and the molecular defect was found in the DCLRE1C gene. She had received 3 doses of OPV. An iVDVP2 with 11 nucleotide differences from Sabin vaccine strain was detected and viral shedding persisted for 3 months until death after a failed BMT.

#### Patient 3

A 10-month-old male born to consanguineous parents presented with unresolving pneumonia, pericardial effusion, draining ears and persistent candidiasis. Screening identified leucopenia, lymphopenia, low CD3 and CD4 levels, and undetectable immunoglobulins. A diagnosis of MHC class II deficiency was made with a homozygous mutation in RFX5 [c.715C>T]. Shortly after diagnosis, he developed AFP and iVDPV2 with 11 nucleotide differences from Sabin vaccine virus was detected and viral shedding persisted for 3 months until the patient died. The patient received 5 doses of OPV, including 3 doses of trivalent OPV and 2 doses of bivalent OPV (containing serotypes 1 and 3).

#### Patient 4

A 6-month-old male infant presented with AFP following the second dose of OPV. Illness started as loss of neck support along with asymmetrical weakness involving both lower limbs, followed by generalized weakness and seizures. Acute disseminated encephalomyelitis (ADEM) was suspected and the patient received intravenous immunoglobulin (IVIG) and steroids. On assessment he was leucopenic and lymphopenic; had low CDs. His IgG was high (just received IVIG) and IgM was low. A diagnosis of T-B+ SCID was made, but the molecular defect was not identified. MRI scanning of the brain showed atrophic frontal lobes with bilateral thalamic patches, judged to be mostly inflammatory in nature. Electromyogram was suggestive of anterior horn cell lesion. The infant also showed a picture of viral myocarditis based on echocardiography with elevated troponin I and mass creatine kinase (MMB). Stool sample revealed iVDPV2 excretion with 1% nucleotide divergence (10 nucleotide differences). Patient died after 3 months.

#### Patient 5

A 14-month-old male presented with AFP associated with disturbed consciousness following OPV. Over 40 days, the patient gradually improved with the left lower limb more affected than the left upper limb, with regain of neck support and ability to sit unsupported. EMG showed evidence of anterior horn cell loss. Following another OPV dose at the age of 9 months, the patient developed progressive right lower limb weakness; both upper limbs were also affected. The clinical condition slightly improved till clinical relapse was reported by the family 15 days after a newborn sibling received OPV. No specimens were collected at that time. On assessment, he had otitis media once. Investigations showed a normal leucocytic count with complete absence of B cells (CD19: 0) and undetectable immunoglobulins. A diagnosis of agammaglobulinemia was made. An iVDPV2 with 17 nucleotide differences from Sabin virus, and SL3 were detected in a stool sample. Viral shedding lasted for 5 months and ceased following IVIG administration.

#### Patient 6

A 22 month-old female, presented with persistent diarrhea, recurrent pneumonia and repeated blood transfusions. One month after her fourth polio dose at the age of 7 months, she developed fever, convulsions, and disturbed consciousness and was admitted with the diagnosis of meningoencephalitis. Investigations showed a low leucocytic count with absolute lymphopenia (ALC: 1656), low CDs and immunoglobulins. A diagnosis of T-B+ SCID was made. Stool sample revealed IVDPV1 with 22 nucleotide differences. The patient died 2 months later.

#### Patient 7

A 9-month-old female, presented with persistent diarrhea, pneumonia and failure to thrive. At the age of 11 months she developed right lower limb hypotonia followed left lower limb affection and progressed to bulbar symptoms followed by coma. The patient received IVIG repeatedly without improvement. EMG and nerve conduction results were consistent with severe diffuse axonal polyneuropathic disease. Family history was positive and investigations revealed CD4 lymphopenia, with undetectable immunoglobulins. There was very defective expression of MHCII on the surface of lymphocytes [HLA DR was 0.6% and CD19+/DR+ was 0.3%]. The diagnosis of MHC Class II deficiency was confirmed by identifying a homozygous missense mutation in RFXANK gene [c.431T>C]. The first stool sample analysis revealed excretion of SL3. Patient died a few weeks thereafter.

### Discussion

This study included PID patients with humoral immunodeficiencies with or without combined cellular immune defects because all patients with poliovirus excretion reported to date have been in these categories [3, 10]. In this study 4.5% of PID patients were identified to be iVDPV excretors which is comparable with 7.3% found in the study conducted among Tunisian patients [3] and much higher than 0.8% found in study from 2014 to 2015 in patients at 19 Jeffrey Model Foundation sites [3].

In our study, iVDPV2 was detected in five of six iVDPV excretors. In 2013, the World Health Assembly endorsed the phased withdrawal of OPV and the introduction of inactivated poliovirus vaccine (IPV) into childhood routine immunization schedules. Type 2 OPV was withdrawn through a globally synchronized “switch” from trivalent OPV (all three types) to bivalent OPV (types 1 and 3) in 155 OPV-using countries in 2016 [11]. Type 2 wild poliovirus was declared eradicated in 2015; however type 2 vaccine virus is the main cause of VDPV outbreaks and approximately 40% of vaccine-associated paralytic polio cases [12].

Most international reports associate iVDPV excretion with patients with antibody deficiency disorders [13, 14]. SCID and combined immunodeficiencies are inherited predominantly in an autosomal recessive manner, thus in inbred populations, the collective results of the AFP surveillance confirmed that the majority of iVDPV cases were among combined immunodeficiency patients rather than among antibody deficiency disorders [15, 16]. As SCID newborn screening is absent in Egypt, the discovery of most SCID patients is delayed after having received several OPV doses. Since bone marrow transplantation therapy is being offered to more patients, they may live longer allowing prolonged virus excretion as with the case reported in Israel [17].

An important PID is the MHC Class II deficiency which has a milder phenotype than SCID patients. It is prevalent in the Middle East region and constitutes an important fraction of patients in populations with high consanguinity as reported by Ben Mustapha et al. [18]. In our study, MHC II deficiency was confirmed in one of the iVDPD2 patients associated with AFP and in the patient with SL3 excretion and AFP. MHC II deficiency patients were also previously reported to demonstrate intermittent iVDPV excretion episodes as well as reinfection with OPV/Sabin like and or non-polio enteroviruses, an observation that necessitates long-term follow up for those patients [3]. Because MHC class II deficiency has a milder phenotype, patients may present later and live longer and pose a public health threat if undiagnosed and shedding virus in the community.

As reported by Driss et al. [19] a Tunisian patient diagnosed with MHC II deficiency exhibited sequential infection by 9 different enterovirus serotypes within a 2-year period. El-Sayed et al. [15] also reported on a PID patient who demonstrated evolution of VDPV in his stool samples over time which again highlights the importance of follow up as they may demonstrate intermittent shedding or evolution with time.

As for humoral defects, patient 5 diagnosed with antibody deficiency cleared virus shedding (iVDPV2 + SL3) following regular institution of IVIG replacement therapy only to relapse (by history) following OPV vaccination of his sibling, emphasizing the implications of contact vaccination for PID families. It is also important to note that AFP can be an initial presentation of PID disorders (patients 4 and 5) and therefore AFP case-patients excreting VDPV should be assessed for immunodeficiency disorders.

### Conclusion

It is critical that paediatricians are aware of the early manifestations that lead to the suspicion of PID, and that they understand the risks associated with the exposure of PID patients to OPV, whether by direct administration or by exposure following OPV vaccination of household contacts. Humoral deficiencies, SCID and CID patients are particularly at risk of developing iVDPV excretion. MHC II deficiency poses a particular risk. Surveillance for prolonged VDPV excretion in countries using OPV is crucial until global cessation of OPV use and the shift towards IPV replacement of OPV vaccination is completed.

## Limitations

No patients with CVID were identified in the study as it was focused on the paediatric age.

## Abbreviations

PID: primary immunodeficiency disorders
IUIS: International Union of Immunological Socities
SCID: severe combined immunodeficiency disorder
AFP: acute flaccid paralysis
XLA: X-linked agammaglobulinemia
iVDPV: immunodeficiency-related vaccine-derived polioviruses
SL: Sabin like virus
MHC class II: major histocompatibility complex II
OPV: oral polio vaccine
BMT: bone marrow transplantation
CDC: Centers for Disease Control and Prevention
CVID: common variable immunodeficiency disorder

## References

[1] Galal N, Meshaal S, Elhawary R, ElAziz DA, Alkady R, Lotfy S, Eldash A, Boutros J, Elmarsafy A (2016). Patterns of primary immunodeficiency disorders among a highly consanguineous population: Cairo University Pediatric Hospital’s 5-year experience. J Clin Immunol.

[2] Reda SM, Afifi HM, Amine MM (2009). Primary immunodeficiency diseases in Egyptian children: a single-center study. J Clin Immunol.

[3] Aghamohammadi A, Abolhassani H, Kutukculer N, Wassilak S, Pallansch M, Kluglein S, Quinn J, Sutter R, Wang X, Sanal O, Latysheva T, Ikinciogullari A, Bernatowska E, Tuzankina I, Costa-Carvalho B, Franco J, Somech R, Karakoc-Aydiner E, Singh S, Bezrodnik L, Espinosa-Rosale FS, Shcherbina A, Lau Y, Nonoyama S, Modell F, Modell V, Barbouche M, McKinlay M, The JMF Centers Network Investigators and Study Collaborators (2017). Patients with primary immunodeficiencies are a reservoir of poliovirus and a risk to polio eradication. Front Immunol.

[4] Shaghaghi M, Soleyman-jahi S, Abolhassani H, Yazdani R, Azizi G, Rezaei N, Barbouche MR, Mckinlay MA, Aghamohammadi A (2018). New insights into pathophysiology of immunedeficiency-associated vaccine-derived poliovirus infection; systematic review of over 5 decades of data. Vaccine.

[5] Ivanova OE, Eremeeva TP, Morozova NS, Shakaryan AK, Gmyl AP, Yakovenko ML, Korotkova EA, Chernjavskaja OP, Baykova OY, Silenova OV, Krasota AY, Krasnoproshina LI, Mustafina AN, Kozlovskaja LI (2016). Vaccine-asociated paralytic poliomyelitis in Russian Federation during the period of changes in vaccination schedule (2006–2013 y.). Vopr Virusol.

[6] Picard C, Al-Herz W, Bousfiha A, Casanova JL, Chatila T, Conley ME, Cunningham-Rundles C, Etzioni A, Holland SM, Klein C, Nonoyama S, Ochs HD, Oksenhendler E, Puck JM, Sullivan KE, Tang ML, Franco JL, Gaspar HB (2015). Primary immunodeficiency diseases: an update on the classification from the international union of immunological societies expert committee for primary immunodeficiency. J Clin Immunol.

[7] Tosato F, Bucciol G, Pantano G, Putti MC, Sanzari MC, Basso G, Plebani M (2015). Lymphocytes subsets reference values in childhood. Cytometry A.

[8] Centers for Disease Control and Prevention. Manual for the surveillance of vaccine-preventable diseases. Centers for Disease Control and Prevention, Atlanta, GA; 2008. https://www.cdc.gov/vaccines/pubs/surv-manual/chpt12-polio.html.

[9] Kew OM, Sutter RW, de Gourville EM, Dowdle WR, Pallansch MA (2005). Vaccine-derived polioviruses and the endgame strategy for global polio eradication. Annu Rev Microbial.

[10] Driss N, Ben-Mustapha I, Mellouli F, Ben Yahia A, Touzi H, Bejaoui M, Ben Ghorbel M, Triki H, Barbouche MR (2012). High susceptibility for enterovirus infection and virus excretion features in Tunisian patients with primary immunodeficiencies. Clin Vaccine Immunol.

[11] Garon J, Seib K, Orenstein WA, Ramirez Gonzalez A, Chang Blanc D, Zaffran M, Patel M (2016). Polio endgame: the global switch from tOPV to bOPV. Expert Rev Vaccines.

[12] Orenstein WA, Committee on Infectious Diseases (2015). Eradicating polio: how the world’s pediatricians can help stop this crippling illness forever. Pediatrics.

[13] Guo J, Bolivar-Wagers S, Srinivas N, Holubar M, Maldonado Y (2015). Immunodeficiency-related vaccine-derived poliovirus (iVDPV) cases: a systematic review and implications for polio eradication. Vaccine.

[14] Macklin G, Liao Y, Takane M, Dooling K, Gilmour S, Mach O, Kew OM, Sutter RW, Diop O, Moeletsi NG, Williams R, The iVDPV Working Group (2017). Prolonged excretion of poliovirus among individuals with primary immunodeficiency disorder: an analysis of the World Health Organization registry. Front Immunol.

[15] El-sayed Z, Mach O, Hossny E, Galal N, El-Sawy I, El-Marsafy A, Reda S, Moussa I, Sibak M, Bassiouni L, Nasr E, Asghar H, Burns C, Chn Q, Oberste M, Sutter R (2016). Poliovirus excretion among persons with primary immunodeficiency disorders: summary of data from enhanced poliovirus surveillance in Egypt, 2011–2014. J Vaccines Vaccin.

[16] Jorba J, Diop OM, Iber J, Sutter RW, Wassilak SG, Burns CC (2016). Update on vaccine-derived polioviruses—worldwide, January 2015–May 2016. MMWR Morb Mortal Wkly Rep.

[17] Weil M, Shulman LM, Heiman S, Stauber T, Alfandari J, Weiss L, Silberstein I, Indenbaum V, Mendelson E, Sofer D (2016). Prolonged excretion of type-2 poliovirus from a primary immune deficient patient during the transition to a type-2 poliovirus-free world, Israel, 2016. Euro Surveill.

[18] Ben-Mustapha I, Ben-Farhat K, Guirat-Dhouib N, Dhemaied E, Larguèche B, Ben-Ali M, Chemli J, Bouguila J, Ben-Mansour L, Mellouli F, Khemiri M, Béjaoui M, Barbouche MR (2013). Clinical, immunological and genetic findings of a large tunisian series of major histocompatibility complex class II deficiency patients. J Clin Immunol.

[19] Driss N, Mellouli F, Ben Yahia A, Touzi H, Barbouche MR, Triki H, Bejaoui M (2014). Sequential asymptomatic enterovirus infections in a patient with major histocompatibility complex class II primary immunodeficiency. J Clin Microbiol.

